# Association between Soy Isoflavone Intake and Breast Cancer Risk for Pre- and Post-Menopausal Women: A Meta-Analysis of Epidemiological Studies

**DOI:** 10.1371/journal.pone.0089288

**Published:** 2014-02-20

**Authors:** Meinan Chen, Yanhua Rao, Yi Zheng, Shiqing Wei, Ye Li, Tong Guo, Ping Yin

**Affiliations:** 1 Department of Epidemiology and Biostatistics, School of Public Health, Tongji Medical College, Huazhong University of Science and Technology, Wuhan, China; 2 WuXiPRA Clinical Research (Shanghai) Co., Ltd, Shanghai, China; 3 Jiangxia Maternal and Child Health Hospital, Wuhan, China; Wayne State University School of Medicine, United States of America

## Abstract

**Background:**

Conclusions drawn from meta-analyses on the association between soy isoflavone intake and breast cancer risk for pre- and post-menopausal women are not fully consistent. These meta-analyses did not explore the influence of different study designs on the pooled results on the basis of distinguishing between pre- and post-menopausal women.

**Methodology and Principal Findings:**

We performed a meta-analysis of 35 studies which reported results of association between soy isoflavone intake and breast cancer risk for pre- and/or post-menopausal women, calculated pooled odds ratios and their 95% confidence intervals of pre- and post-menopausal women respectively, and further explored soy isoflavone-breast cancer association on the basis of considering different study regions and designs. Summary results suggested that soy isoflavone intake has a protective effect against breast cancer for both pre- and post-menopausal women. However, they are influenced by study design and region. Pooled ORs of studies carried out in Asian countries suggested that soy isoflavone’s protective effect exist in both pre- and post-menopausal women (OR = 0.59, 95%CI: 0.48–0.69 for premenopausal women; OR = 0.59, 95%CI: 0.44–0.74 for postmenopausal women). However, there are some differences between the results pooled from different study designs for women in Asian countries (test for consistency, *P* = 0.04). Pooled OR of studies on postmenopausal women in Western countries suggested that soy isoflavone intake has a marginally significant protective effect (OR = 0.92; 95%CI: 0.83∼1.00), but further analyses stratifying by study design found no statistically significant association.

**Conclusions:**

We meta-analyzed more and newer research results, and separated women according to menopausal status to explore soy isoflavone-breast cancer association. We founded that soy isoflavone intake could lower the risk of breast cancer for both pre- and post-menopausal women in Asian countries. However, for women in Western countries, pre- or post-menopausal, there is no evidence to suggest an association between intake of soy isoflavone and breast cancer.

## Introduction

Epidemiologic studies have explored the association between soy isoflavone intake and breast cancer risk since Lee HP et al. [1] reported that soy protein may lower breast cancer risk for premenopausal women. However, these findings are highly variable, especially on soy isoflavone-breast cancer association drawn for pre- and post-menopausal women. Meta-analyses published so far have not drawn a consistent conclusion. Bruce JT et al. [2] concluded that the protective effect of soy isoflavone was somewhat stronger in premenopausal women than in postmenopausal women. However, AH Wu et al. [3] have reported that menopausal status may has no modifying effect on the soy-breast cancer association (a significant inverse association was observed in both pre- and post-menopausal women.), and meta-analyses by Dong JY et al. [4] and Zhong X et al. [5] suggested that the protective effect of soy isoflavone (or soy food) intake for breast cancer exists among postmenopausal women only. Results of some Randomized Controlled Trials [6] indicated that isoflavone has a different impact on pre- and post-menopausal women’s mammary density. Lee H et al. [6] meta-analyzed these RCTs’ results and concluded that isoflavone intake does not alter the mammary density in postmenopausal women, but may slightly increase it in premenopausal women.

When explored the soy isoflavone-breast cancer association stratified by menopausal status, previous meta-analyses rarely further considered the influence of different study regions and designs. Given the inconsistence of these results, we conducted an up-to-date meta-analysis of the soy isoflavone-breast cancer association in pre- and post-menopausal women, examining the association (pooled odds ratios) stratified by study region and soy isoflavone exposure measurement et al., and fully took study design’s influence on these pooled results into consideration, aiming at exploring the association between soy isoflavone intake and breast cancer risk for pre- and post-menopausal women systematically.

## Methods

### Ethical Consideration

This study was reviewed and approved by the ethical committee of School of Public Health, Tongji Medical College, Huazhong University of Science and Technology, Wuhan, China.

### Search Strategy

The PRISMA checklist is available as an appendix (**Checklist S1**). An electronic literature search of PubMed and Web of Science was conducted using the following search terms: isoflavone (including phytoestrogen, daidzein, genistein), soy (including bean, soybean, soyfood, tofu, miso), breast cancer and menopausal (including premenopausal, postmenopausal, perimenopausal). All publications up to January 2013 were considered. Simultaneously, we reviewed citations listed in retrieved articles to identify additional studies. No attempt was made to obtain unpublished data.

### Study Selection Criteria

We set the following criteria to select eligible studies:

1. Study design- cohort study, nested case-control or case-control study;
2. Soy isoflavone intake was defined as intake of isoflavone (including genistein and daidzein), or soy protein, or soy foods/products (including tofu, soybean, lentils, miso), or using isoflavone level in urine/plasma as the exposure measurement;
3. The study endpoint was breast cancer incidence (only for cohort studies);
4. Studies reported Odds Ratio/Relative Risks and their corresponding 95% confidence intervals quantifying the association between soy isoflavone intake and breast cancer risk for pre- and/or post-menopausal women.

Two reviewers searched literature with the same retrieval strategy independently, assessed retrieved titles and abstracts, and downloaded potentially relevant articles for further assessment of inclusion. If multiple papers reported results from the same study cohort, whether research participants overlapped (if so, the number of participants), degree of conformance to the predefined inclusion criteria, and publication year were comprehensively reviewed to select the appropriate papers for the analysis.

### Data Extraction

The same two reviewers read full-text articles independently, and extracted the following information from the original articles: name of the first author, publication year, study region (country area), study design (cohort or case-control study), number of cases and controls or person-years, exposure measurement (soy isoflavone), the most adjusted odds ratios(OR) in case-control studies or relative risk (RR) in cohort studies and the corresponding 95% confidence intervals (CI) comparing the highest vs. lowest (or reference) level of soy isoflavone intake, and the adjustment factors in multivariable models. Disagreements on data extraction were resolved through discussion.

### Statistical Analysis

The pooled ORs estimating the association between soy isoflavone intake and breast cancer risk for pre- and post-menopausal women separately were calculated. If multiple exposure measurements of soy isoflavone were reported to evaluate the association with breast cancer risk, the priority order of isoflavone, soy protein, soy foods/soy products (including soy bean and tofu et al.) was taken in summary analyses. In subgroup analyses (stratified by exposure measurements), we took full advantage of reported results as needed.

Q test was used to test the homogeneity of results across studies, and *I^2^* was calculated to quantify the heterogeneity among studies, if *P*<0.10, it means that there was significant heterogeneity, and random-effects model was used to obtain the pooled estimates. Otherwise, fixed-effects model was applied.

In order to make comparisons between pooled estimates obtained from different study designs, we calculated the pooled RR (OR_1_) in cohort or nested case-control studies and retrospective case-control studies (OR_2_) for all predefined subgroups. The ratio of OR_1_/OR_2_ (OR_C_) was calculated and *z*-statistic [7] was used to test whether the difference between OR_1_ and OR_2_ is significant.

We deleted studies that limited soy isoflavone intake to special period or measured soy isoflavone intake with a significantly different method from others (details in results section). We performed sensitive analyses to evaluate the robustness of summary results.

Potential publication bias was examined with Egger method, which implements a weighted linear regression of the logarithm of the odds ratios on their standard errors, simultaneously, funnel plots were given for a visual inspection on publication bias. If *P* value obtained from Egger test was <0.10, then we considered that potential publication bias exists.

## Results

### 1 Description of Included Studies

**Figure 1:**
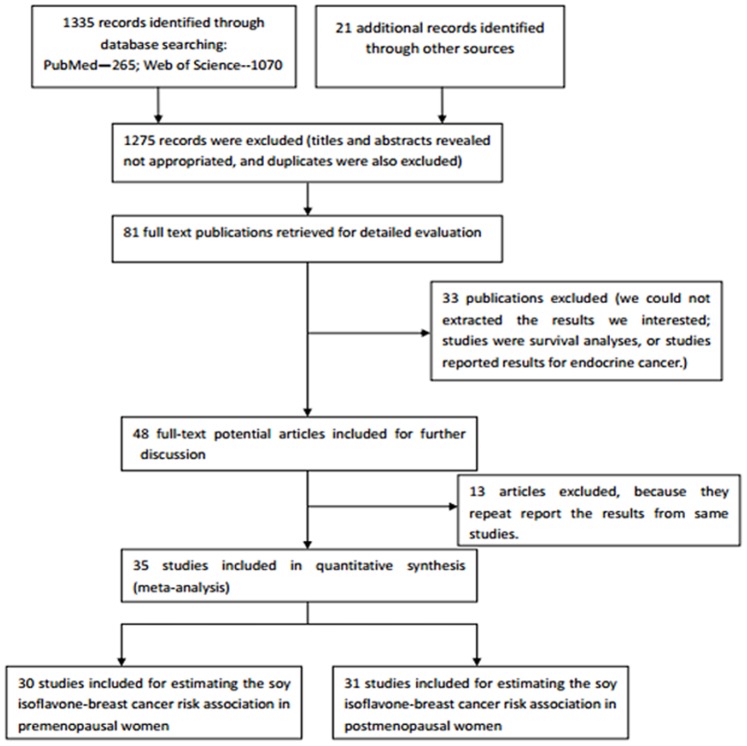
Flowchart illustrating movement of papers from search to inclusion.

1335 citations were retrieved by electronic searching, and 23 articles were identified by bibliographic searching. 84 articles were potentially relevant after titles and abstracts were read. Among them, 36 articles did not meet the predefined includsion criteria after reviewing the full paper by two independent reviewers, and 13 articles were excluded for repetitive reporting results from the same studies. Finally, 35 studies were included in the meta-analysis, including 26 studies reporting the association between soy isoflavone intake and breast cancer risk for both pre- and post-menopausal women, 4 studies reporting the association in premenopausal women only, and the remaining 5 studies in postmenopausal women only. Therefore, results from 30 studies were included to assess the soy isoflavone-breast cancer association among premenopausal women, and 31 studies were included for assessing the association in postmenopausal women. **Figure 1** presents the flowchart of the study selection process.

Of the 30 studies assessing the association between soy isoflavone intake and breast cancer risk in premenopausal women, 10 studies are cohort or nested case-control study (4 studies in Asian countries. 6 studies in Western countries) and 20 studies are case-control study (13 studies in Asia, and 8 studies in Western countries). Of the 31 studies on postmenopausal women, 12 are cohort or nested case-control studies (5 studies in Asia, and 7 in Western countries), and 19 are case-control studies (13 studies in Asian countries, 7 in Western countries). Most studies included the results adjusted for a wide range of potential confounding factors, including age, alcohol and smoking, body mass index (BMI), energy intake and hormone replacement therapy. **Table S1** shows the characteristics of the included studies, and the other two excel documents named “**Excel S1**” and “**Excel S2**” respectively present pre- and post-menopausal women’s information with different sheets displaying data information of specific study characteristics (Asian/Western/earlier/later).

There is something needed special attention: two studies [8]–[9] gave results stratified by receptor type. Laura NA [8] reported results of ER+/PR+, ER−/PR− and ER+/PR− three receptor types, Min Z [9] stratified receptor type according to ER and PR status and reported results for ER+, ER−, PR+ and PR− respectively. For the latter, we extracted results of ER+ and ER−. Therefore, we take [8] as three and [9] as two separated studies to make meta-analysis. Similarily, Motoki I et al. [10] carried out their study in Japan and Brazil, and they reported results of pre- and post-menopausal women who were Japanese, Japanese living in Brazil and non-Japanese living in Brazil apart, since study region is a stratified factor we predefined, we also included Motoki I et al. [10] as three individual studies for analysis.

### 2 Summary and Subgroup Analyzed Results for Pre- and Post-menopausal Women Respectively

(**Table 1** presents pooled OR estimates from summary and stratified analysis.).

**Table 1:** Pooled odds ratios (ORs) and 95% confidence intervals (CIs) for high versus low soy isoflavone intake (as defined in each original study) associating with breast cancer risk, calculated from summary and stratified (by predefined factors) analyses.

Group	No. of studies (cases)	Heterogeneity of ORs					OR (95%CI)
		*χ^2^*(*df*Λ)	*P* *		*I^2^*(%)		
**Premenopausal**							
Summary	30(10888)	107.33(34)		0.000		68.3	0.74(0.64–0.85)
AsianΨ	17(5466)	36.32(17)		0.004		53.2	0.59(0.48–0.69)
WesternΨ	14(5422)	35.51(16)		0.003		54.9	0.90(0.77–1.04)
Soy isoflavone$/protein	22(6710)	98.44(26)		0.000		73.6	0.76(0.62–0.89)
Soy bean/soy products(foods)#	11(4702)	29.87(10)		0.001		66.5	0.64(0.49–0.80)
Earlier time@	12(5027)	25.26(11)		0.008		56.5	0.74(0.59–0.88)
Later time&	18(5861)	81.98(22)		0.000		73.2	0.75(0.61–0.89)
**Postmenopausal**							
Summary	31(16705)	223.33(35)		0.000		84.3	0.75(0.63–0.86)
AsianΨ	18(4581)	93.28(18)		0.000		80.7	0.59(0.44–0.74)
WesternΨ	14(12124)	25.20(16)		0.066		36.5	0.92(0.83–1.00)
Soy isoflavone$/protein	21(9341)	205.48(25)		0.000		87.8	0.73(0.58–0.88)
Soy bean/soy products(foods)#	13(7727)	138.77(12)		0.000		91.4	0.72(0.48–0.97)
Earlier time@	10(5002)	25.55(9)		0.002		64.8	0.76(0.59–0.94)
Later time&	21(11703)	197.66(25)		0.000		87.4	0.74(0.60–0.88)

* *P* values (two-sided) were based on the *Q* test of heterogeneity.

Λ Degree of freedom (*df*) dose not equal N-1 because three studies’ data [8]–[10] were extracted as three or two independent studies.

@Before 2006.

& Since 2006.

$ Soy isoflavone includes exposure measured by dietary isoflavone intake, plasma genistein concentration, or urinary isoflavone excretion.

# Soy bean/soy products includes soybeans, soy, beans or tofu.

Ψ The study [10] was carried out in both Japan and Brazil by Motoki Iwasaki et al., its data was retained as three individual studies for analysis, hence larger total number of studies stratified by study region than summary studies.

#### 2.1 Association of soy isoflavone intake and breast cancer risk among premenopausal women

**Figure 2:**
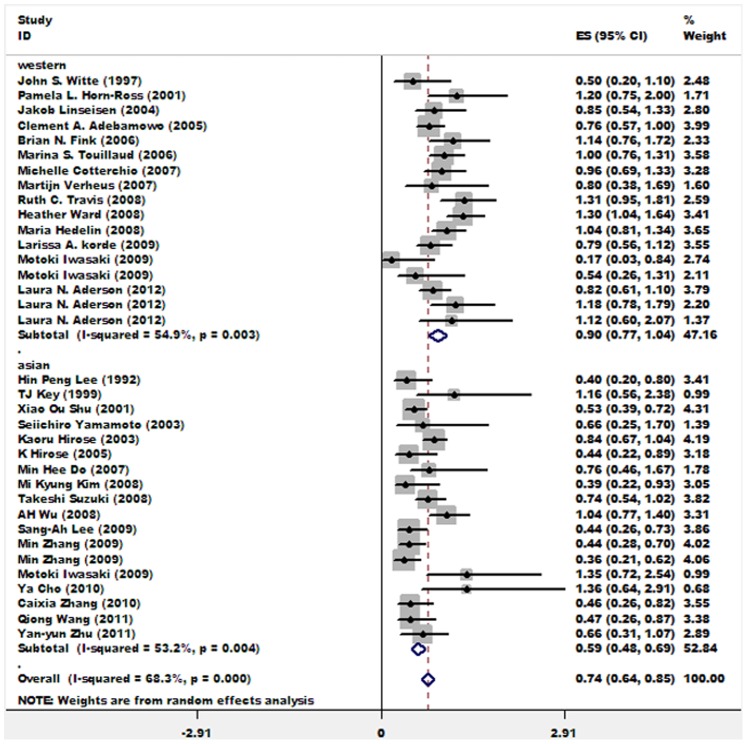
Associations between soy isoflavone intake and breast cancer risk in all studies and studies carried out in Asian or Western countries among premenopausal women. Relative weights are indicated by the area of square. Horizontal lines represent 95% confidence intervals for the odds ratios.

Summary results of 30 studies exploring the soy isoflavone-breast cancer association for premenopausal women indicates that soy isoflavone intake was inversely associated with breast cancer risk (OR = 0.74; 95%CI: 0.64∼0.85). When stratified by study region, 17 studies carried out in Asian countries suggested that soy isoflavone was protective in premenopausal Asian women to some extent, while 14 studies in Western nations didn’t obtain a statistically significant association (OR = 0.90; 95%CI: 0.77∼1.04). **Figure 2** shows the forest plot presenting soy isoflavone-breast cancer association in premenopausal women. The word document entitled “**Forest Plots S1**” presents forest plots with weights for subgroup analyses (among premenopausal women) which were further stratified by study design, meanwhile, heterogeneity statistics for each stratified analysis were listed below each forest plot.

Then we stratified these 30 studies by measurement of soy isoflavone and publication year. ORs and their 95% CIs pooled from 22 studies using soy isoflavone or soy protein as intake measurement and 11 studies using that of soy bean/soy products were (OR = 0.76; 95%CI: 0.62∼0.89) and (OR = 0.64; 95%CI: 0.49∼0.80) respectively, both suggested that soy isoflavone had a protective effect in premenopausal women, while the latter was somewhat stronger. Similar pooled ORs were observed in 18 recently published studies (from 2006 to January 2013) and 12 earlier studies (before 2006). (**Table 1**).

#### 2.2 Association of soy isoflavone intake and breast cancer risk among postmenopausal women

**Figure 3:**
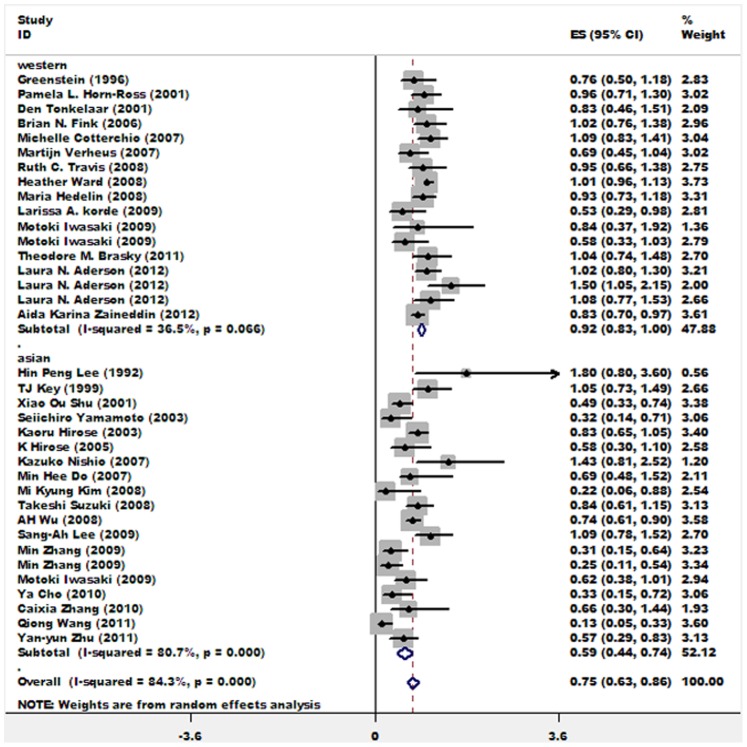
Associations between soy isoflavone intake and breast cancer risk in all studies and studies carried out in Asian or Western countries among postmenopausal women. Relative weights are indicated by the area of square. Horizontal lines represent 95% confidence intervals for the odds ratios.

Pooled result from 31 studies estimating the association between soy isoflavone intake and breast cancer risk among postmenopausal women indicates that highest intake of soy isoflavone vs. lowest intake could decrease breast cancer risk by about 25% for postmenopausal women (OR = 0.75; 95%CI: 0.63∼0.86). When stratified by study region, 18 studies carried out in Asian countries pooled a result suggesting an inverse soy isoflavone-breast cancer association (OR = 0.59; 95%CI: 0.44∼0.74), while a weak but statistically significant inverse association was found among postmenopausal women living in Western nations (OR = 0.92; 95%CI: 0.83∼1.00). **Figure 3** shows the forest plot of OR/RR estimates for postmenopausal women. The word document entitled “**Forest Plots S2**” presents forest plots with weights for subgroup analyses (among postmenopausal women) which were further stratified by study design, meanwhile, heterogeneity statistics for each stratified analysis were listed below each forest plot.

Similarly, we stratified these 31 studies according to measurement of soy isoflavone intake and publication year and pooled OR estimates. 21 studies used soy isoflavone or soy protein as intake measurement, and 13 studies used soy bean or soy products. Pooled ORs and their 95% CIs were OR = 0.73; 95%CI: 0.58∼0.88 and OR = 0.72; 95%CI: 0.48∼0.97, respectively. Both of them suggested a protective effect of soy isoflavone to postmenopausal women. Earlier studies (before 2006) and later studies (from 2006 to January 2013) obtained similar pooled ORs. (**Table 1**).

### 3. Further Analyze the above Results Stratified by Study Design

(**Table 2** shows further stratified results and comparison odds of each pair).

**Table 2:** Comparison of soy isoflavone-breast cancer association among studies by study design (cohort or nested case-control versus. retrospective case-control studies).

Comparison groups	Cohort or nested case-control study		Retrospective case-control study		Comparison OR_C_Ψ(95%CI)	*P* **
	No. of studies	OR_1_$ (95%CI)	No. of studies	OR_2_& (95%CI)		
Premenopausal						
Summary	10	0.94(0.74–1.14)	20	0.66(0.55–0.76)	1.42(1.09–1.87)	0.01
Asian	4	0.77(0.37–1.18)	13	0.56(0.45–0.66)	1.38(0.75–2.53)	0.31
Western	6	1.03(0.84–1.22)	8	0.81(0.64–0.99)	1.27(0.95–1.69)	0.10
Soy isoflavone/protein	8	0.96(0.72–1.21)	14	0.65(0.51–0.79)	1.48(1.05–2.07)	0.02
Soy bean/soy products(foods)	2	0.78(0.57–0.99)	9	0.61(0.44–0.78)	1.28(0.86–1.90)	0.23
Earlier time	4	0.85(0.69–1.01)	8	0.68(0.50–0.87)	1.25(0.89–1.75)	0.19
Later time	6	0.99(0.67–1.30)	12	0.64(0.51–0.77)	1.55(1.05–2.29)	0.03
Postmenopausal						
Summary	12	0.86(0.73–1.00)	19	0.69(0.54–0.83)	1.25(0.95–1.63)	0.11
Asian	5	0.84(0.54–1.14)	13	0.50(0.34–0.66)	1.68(1.02–2.77)	0.04
Western	7	0.96(0.88–1.04)	7	0.92(0.78–1.06)	1.04(0.88–1.24)	0.63
Soy isoflavone/protein	8	0.82(0.65–0.99)	13	0.69(0.49–0.88)	1.19(0.83–1.70)	0.35
Soy bean/soy products(foods)	4	0.96(0.76–1.17)	9	0.60(0.32–0.89)	1.60(0.92–2.79)	0.10
Earlier time	4	0.72(0.38–1.06)	6	0.80(0.58–1.01)	0.90(0.50–1.61)	0.72
Later time	8	0.92(0.79–1.04)	13	0.65(0.47–0.82)	1.42(1.04–1.93)	0.03

$ Pooled OR of cohort or nested case-control studies.

& Pooled OR of retrospective case-control studies.

Ψ The comparison odds ratio, is the ratio of OR_1_ versus OR_2_. OR_C_>1 implies that studies employed prospective design (cohort or nested case-control study) exhibited a weaker protective association than studies employed retrospective design (case-control study). Conversely, OR_C_<1 implies that studies employed prospective study design is associated with stronger protective associations.

**P values (two-sided) were calculated using the *Z*-statistic [7].

#### 3.1 Stratified analyses and comparisons for premenopausal women

Of 30 studies assessing soy isoflavone intake-breast cancer association in premenopausal women, 10 were cohort or nested case-control studies, and 20 were retrospective case-control studies. The pooled OR estimates obtained from cohort or nested case-control studies did not suggest an association between soy isoflavone intake and breast cancer (OR = 0.94; 95%CI: 0.74∼1.14), while the pooled OR from case-control studies hinted a protective effect of soy isoflavone to premenopausal women (OR = 0.66; 95%CI: 0.55∼0.76). Difference between these two pooled results was statistically significant, as indicated by OR_C_ (pooled OR of cohort or nested case-control studies versus that of retrospective case-control studies).

4 cohort and 13 case-control studies carried out in Asian area obtained pooled ORs of 0.77, 95%CI: 0.37∼1.18 and 0.56, 95%CI: 0.45∼0.66, respectively. The former didn’t suggest an inverse association between soy isoflavone intake and breast cancer risk, but the latter did. And the difference between these two had no statistically significance (OR_C_ = 1.27; 95%CI: 0.95∼1.96; *P* = 0.10). Studies in Western countries obtained similar results.

2 cohort studies and 9 case-control studies using soy bean/soy products as intake measurement suggested an inverse association between soy isoflavone intake and breast cancer risk, their pooled ORs were OR = 0.78; 95%CI: 0.57∼0.99 and OR = 0.61; 95%CI: 0.44∼0.78 respectively. However, 8 cohort studies using soy isoflavone didn’t suggest any association, but case-control studies did. And the difference between these two had statistically significance (OR_C_ = 1.48; 95%CI: 1.05∼2.07; *P* = 0.02). In addition, for studies published from 2006 to most recently, case-control studies was associated with a stronger protective associations than cohort studies (OR_C_ = 1.55; 95%CI: 1.05∼2.29; *P* = 0.03).

#### 3.2 Stratified analyses and comparisons for postmenopausal women

12 cohort studies and 19 case-control studies exploring the soy isoflavone-breast cancer association among postmenopausal women obtained pooled ORs of 0.86, 95%CI: 0.73∼1.00 and 0.69, 95%CI: 0.54∼0.83, respectively. Both suggested a protective effect of soy isoflavone for postmenopausal women, and the difference between them was not statistically significant (OR_C_ = 1.25; 95%CI: 0.95∼1.63; *P* = 0.11). However, we must be cautious of the significant inverse association pooled from all cohort studies: when we stratified 12 cohort studies by publication year or study region, all the 4 pooled ORs were not statistically significant (“cohort study” column in table 2).

For women residing in Asian area, difference between two pooled ORs analyzed from 5 cohort studies (OR = 0.84; 95%CI: 0.54∼1.14) and 13 case-control studies had statistically significantly difference (OR_C_ = 1.68; 95%CI: 1.02∼2.77; *P* = 0.04). Here, case-control studies hinted a strong inverse association between soy isoflavone intake and breast cancer risk (OR = 0.50; 95%CI: 0.34∼0.66). Pooled OR of 7 cohort studies and that of 7 case-control studies carried out in Western countries didn’t suggest an association. These results warned us that we should reassess the weak but statistically significant inverse association among Western postmenopausal women reported in section 2.2.

8 cohort studies and 13 case-control studies using soy isoflavone as intake measurement suggested an inverse association between soy isoflavone intake and breast cancer risk, their pooled ORs were OR = 0.82; 95%CI: 0.65∼0.99 and OR = 0.69; 95%CI: 0.49∼0.88 respectively. However, 4 cohort studies using soy bean/soy products didn’t suggest any association. In addition, when studies published later (since 2006 to January 2013) were stratified by study design, the statistically significant difference between two pooled ORs suggested case-control studies was associated with a stronger protective associations (OR_C_ = 1.42; 95%CI: 1.04∼1.93; *P* = 0.03).

### 4 Results of Sensitive Analyses

#### 4.1 Sensitive analysis for premenopausal women

Xiao OS [11] limited intake period of soy foods to adolescence and assessed its association with subsequent breast cancer risk. Martijn V [12] and Heather W [13] used plasma genistein and total urinary isoflavones as intake measurements to evaluate their association with breast cancer risk, which were different from the other included studies. Therefore, we deleted these three studies in a sensitive analysis. Heterogeneity among studies decreased a little bit (*I* ^2^ = 64.7%) but remained, thus we used random effects model, and pooled OR and its corresponding 95%CI were 0.73(0.63∼0.83), which was very close to overall summary pooled OR.

#### 4.2 Sensitive analysis for postmenopausal women

Besides 3 studies [11]–[13] mentioned above, Theodore MB et al. [14] carried out their study in America, and explored whether soy supplement intake was associated with postmenopausal women’s breast cancer risk. Similar to studies reported by Martijn V [12] and Heather W [13], Isoide DT [15] reported odds ratio for urinary genistein excretion in association with breast cancer risk for postmenopausal women. Hence, 5 studies [11]–[15] were excluded when employed in a sensitive analysis for postmenopausal women. We found that heterogeneity changed a little (*I* ^2^ = 64.7%), and random effect model was used to obtain pooled a OR of 0.74(95%CI: 0.61∼0.86), which was also similar to the overall summary pooled OR.

### 5 Tests for Publication Bias

#### 5.1 Publication bias for soy isoflavone intake in association with breast cancer risk in premenopausal women

**Figure 4:**
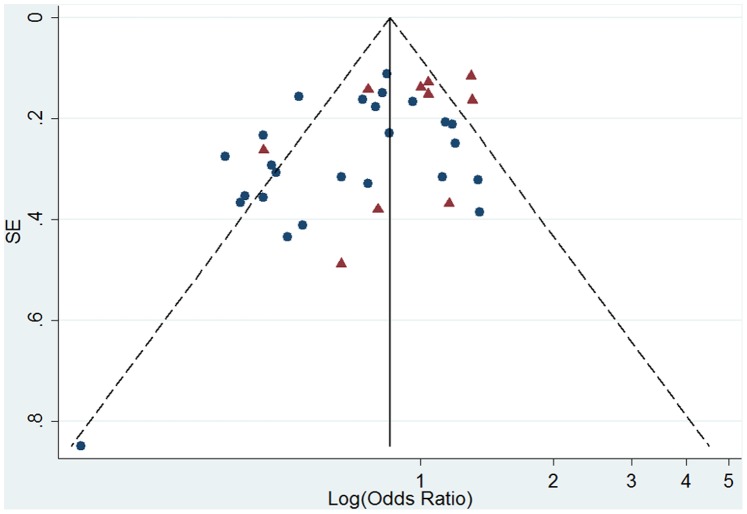
Funnel plot of log (odds ratio) for high versus low soy isoflavone intake and standard error (SE) of log (odds ratio) among premenopausal women. Triangle, studies carried out in western nations; circle, studies carried out in Asia.

Egger regression test was carried out for 30 studies included in the premenopausal women analysis, and a significant publication bias was observed (intercept = −1.83, 95%CI = −3.14∼−0.51; *P* = 0.008). And funnel plot also showed an asymmetrical distribution (**Figure** **4**). Then we employed Egger regression test for Asian studies and Western studies separately. Potential publication bias was found among Western studies (*P* = 0.044), but not among Asian studies (*P* = 0.202).

#### 5.2 Publication bias for soy isoflavone intake in association with breast cancer risk in postmenopausal women

**Figure 5:**
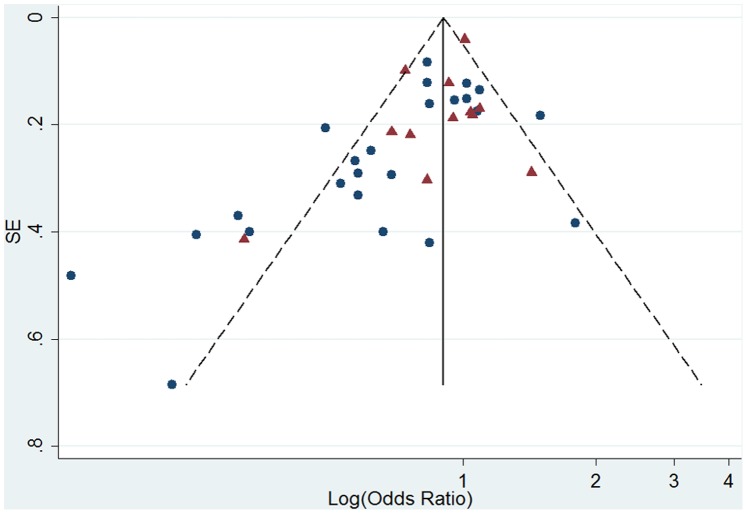
Funnel plot of log (odds ratio) for high versus low soy isoflavone intake and standard error (SE) of log (odds ratio) among postmenopausal women. Triangle, studies carried out in western nations; circle, studies carried out in Asia.

Visually, the funnel plot had an asymmetrical distribution (**Figure 5**). Egger regression test suggested a potential publication bias in 31 studies included in meta-analysis for postmenopausal women (intercept = −1.58, 95%CI = −2.45∼−0.72, *P* = 0.001). Further Egger tests stratified by study region obtained results opposite to premenopausal women’s: publication bias was not found among Western studies (*P* = 0.200), but observed among Asian studies (*P* = 0.027).

## Discussion

Soy foods or soy products are rich source of isoflavone, which presents both weak estrogenic and anti-estrogenic effects [1], [16]. Premenopausal women are greatly different from postmenopausal women in endogenous estrogen level, and that breast cancer is a disease closely associated with hormone. Therefore, menopausal status may play a modifying role in soy isoflavone-breast cancer association [4]. Recently, abundant studies in this field are constantly emerging, and more and more studies set premenopausal women apart from postmenopausal women and reported OR/RR estimates for breast cancer risk associated with soy isoflavone intake respectively. Laura NA et al. [8] reported results gained from a population based case-control study: adult isoflavone intake had no effects on the incidence of breast cancer for both pre- and post-menopausal women. Moreover, they concluded that adult isoflavone intake was positively associated with ER−/PR− tumors among postmenopausal women. Considering the inconsistence of results in a number of studies, it appears essential to summarize more and newer study results in understanding the soy isoflavone-breast cancer association for pre- and post-menopausal women.

Our meta-analysis included 35 studies. There were 30 studies’ results for premenopausal women, and 31 studies for postmenopausal women. Summary results suggested that high soy isoflavone intake presents some protective effects on breast cancer for both pre- and post-menopausal women. However, we must be cautious, because results were influenced by study design, especially for premenopausal women. Pooled ORs of cohort studies were statistically different from pooled ORs of case-control studies, the latter hinted a strong protective effect of soy isoflavone in premenopausal women, but cohort studies didn’t obtain a statistically significant association. Previous meta-analyses [3], [5], [17] drew similar conclusions when stratified the analyses by study design without separating pre- and post-menopausal women apart, and they didn’t make comparisons between pooled OR estimates from studies of different designs (cohort or case-control study). There is great difference between prospective cohort and retrospective case-control studies. Relatively speaking, case-control studies inevitably suffer some drawbacks like recall and selection biases. Therefore, pooled results which did not stratified by study design are debatable, especially for those ORs which are very close to 1. Pooled results of studies published earlier (before 2006) evaluating soy isoflavone intake’s association with postmenopausal women’s breast cancer risk and pooled result for Western postmenopausal women both corroborated the above opinion. Stratifying pooled results by study design were intended to check whether corresponding results will be biased by study design.

Published 4 meta-analyses [3]–[5], [17] all concluded that soy isoflavone/soy food intake was inversely associated with breast cancer risk among Asian women, but this association did not exist among Western women. Our meta-analysis drew a conclusion that an inverse association between soy isoflavone and breast cancer risk existed among both pre- and post-menopausal women in Asian. However, in terms of Asian postmenopausal women, the difference between pooled OR estimates of studies stratified by study design was statistically significant, suggesting that difference in study design contributed to heterogeneity among studies. Although the pooled OR estimate for postmenopausal women in Western nations suggested a protective effect of soy isoflavone, the protective effect disappeared when stratified by study design. This indicated that drawing a conclusion that soy isoflavone intake could lower breast cancer risk for Western postmenopausal women may be not so reliable. The weak protective effect could be just a result from statistical chance finding. It appeared that soy isoflavone intake had no influence on breast cancer risk among pre- and post-menopausal women in Western countries. Exposure to soy isoflavone in early life and high intake level may be important contributing elements for its protective effect to Asian women (pre- and post-menopausal). And non-association between soy isoflavone intake and breast cancer risk among Western pre-and post-menopausal women may be related to their low intake levels of soy isoflavone which couldn’t come into play yet in low dose [18].

Some studies[1], [9], [19]–[23] reported ORs/RRs for breast cancer risk in association with multiple assessments of soy isoflavone exposure simultaneously. We found that pooled OR estimates of results using soy isoflavone/soy protein as exposure measurement were very close to summary results for both pre- and post-menopausal women. However, for premenopausal women, pooled ORs of soybean/soy foods intake and breast cancer risk was different from summary result. As for the reason, we consider that most of the included studies took isoflavone as an intake measurement, but some studies using soybean/soy products (along with soy isoflavone) as intake measurements as additional results in subgroup analyses. Furthermore, for studies using soy isoflavone/soy protein as exposure measurements, both pooled estimates of cohort studies and case-control studies hinted a protective association between soy isoflavone intake and breast cancer risk among postmenopausal women. The influence to pooled results caused by difference in exposure measurements cannot be ignored. Future studies in this field should quantify isoflavone as accurate as possible with comprehensive soy foods items to evaluate its association with breast cancer risk. Although isoflavone may be the major substance impacting breast cancer risk, we can’t assert that there is nothing else work similarly (as far as we know, like lignant.). In addition, the amount of isoflavone varies greatly among different soy foods. if we assess soy foods intake generally and take it as isoflavone without measurement transformation to estimate its association with breast cancer risk, the inaccuracy of intake measurement is inevitable. This may be an important source of heterogeneity.

Although our method of estimating pooled OR was a little different from Trock et al. [2], we found that pooled OR estimates of studies published before 2006 (OR = 0.74; 95%CI: 0.59∼0.88 for premenopausal women and OR = 0.76; 95%CI: 0.59∼0.94 for postmenopausal women) were similar to pooled ORs of Trock et al. [2] (OR = 0.70; 95%CI: 0.58∼0.85 for premenopausal women and OR = 0.77; 95%CI: 0.60∼0.98 for postmenopausal women). The inverse association between soy isoflavone intake and breast cancer risk was somewhat stronger in premenopausal women than in postmenopausal women. However, we must prudently treat pooled results of postmenopausal women (published before 2006): pooled result of neither cohort nor case-control studies suggested a significant inverse association. So, the protective effect of soy isoflavone in postmenopausal women (concluded in 2006) appears to be questionable. This, once again, proved that it is necessary to employ stratified analyses by study design in summary comparisons. In addition, pooled ORs of later published cohort studies (since 2006) didn’t suggest a protective effect of soy isoflavone, and the differences between pooled ORs of studies of different designs were statistically significant. Hence we can see that the association between soy isoflavone intake and breast cancer risk is influenced by study design to a large extent.

Three studies [12]–[13], [15] used urine or plasma isoflavone level to assess exposure, these methods avoided measurement inaccuracy in using food frequency questionnaire to evaluate low dietary soy isoflavone intake, but urinary/plasma isoflavone level could just reflect short-term intake status of participants, hence the randomness couldn’t be ignored. Thus it couldn’t represent dietary intake level and may be a major source of nondifferential measurement errors. Together with two studies [11], [14] limited to soy isoflavone intake in adolescence [11] or soy supplement intake [14], we deleted these 5 studies in sensitive analyses, and found that pooled OR estimates changed little for both pre- and post-menopausal women, demonstrating the robustness of our summary pooled results.

We avoid mistakes reported by Allyson Delaune et al. [24] in their critical appraisal paper concerning the credibility of a meta-analysis about the role of dietary soy intake in reducing breast cancer risk. Still, some limitations exist in our meta-analysis should be cautioned: heterogeneity among studies, diversity in intake measurements, and potential publication bias. Besides, we only compared the estimates of highest vs. lowest groups of soy isoflavone intake for breast cancer risk. Further dose-response relationship should be analyzed to more accurately estimate soy isoflavone-breast cancer risk association in future studies. Actually, in a recently published paper, Qi Xie et al. [25] conducted a dose-response meta-analysis of observational studies, and they drew similar conclusions as we did, which further strengths the credibility of our conclusions to some extent.

And it is noteworthy that two newly published articles [26]–[27] concerned the association between soy food intake after cancer diagnosis with breast cancer survival. Although the endpoints and the study populations of these two studies are different from our study, their conclusions are very inspiring. Their findings further confirmed the protective effect of soy isoflavone for breast cancer. In additional, Feng Chi et al. [26] also concluded that menopausal status could influence the association between soy food intake after cancer diagnosis with breast cancer recurrence. It, once again, proved the modifying effect of menopausal status, and showed that separating pre- and post-menopausal women to explore the soy isoflavone-breast cancer risk association might be the right approach and something has to be taken into consideration.

In summary, our meta-analysis shows that soy isoflavone presents a protective effect for Asian pre- and post-menopausal women to some extent, but not for Western women. Further stratified and compared results (taking study design to fully consideration) gave us warnings: when we treat pooled estimates, adequate attention should be paid to study design’s influence: pooled ORs from case-control studies with lower demonstrate strength tend to suggest an inverse association between soy isoflavone intake and breast cancer risk. However, pooled ORs from cohort studies hardly indicate soy isoflavone’s effect on breast cancer. Therefore, pooled results regardless of study design may be biased to some extent. For these reasons, we shouldn’t be arbitrary when draw conclusions according to pooled results, especially for some marginally significant effect.

## Supporting Information

Table S1 Characteristics of included epidemiological studies exploring the association between soy isoflavone intake and breast cancer risk.
(DOCX)

Checklist S1 PRISMA checklist.
(DOC)

Excel S1 The excel file presents premenopausal women’s data information extracted from studies of specific characteristics (sheet names indicate the study characteristics).
(XLSX)

Excel S2 The excel file presents postmenopausal women’s data information extracted from studies of specific characteristics (sheet names indicate the study characteristics).
(XLSX)

Forest Plots S1 The word file presents six forest plots for subgroup analyses among premenopausal women, and in each analysis, studies were further stratified by study design, meanwhile, heterogeneity statistics for each stratified analysis were listed below each forest plot.
(DOCX)

Forest Plots S2 The word file presents six forest plots for subgroup analyses among postmenopausal women, and in each analysis, studies were further stratified by study design, meanwhile, heterogeneity statistics for each stratified analysis were listed below each forest plot.
(DOCX)
