# Supplementary material for: Association between Soy Isoflavone Intake and Breast Cancer Risk for Pre- and Post-Menopausal Women: A Meta-Analysis of Epidemiological Studies
Source: PLoS One. 2014 Feb 20;9(2):e89288. doi: 10.1371/journal.pone.0089288 (PMC3930722; doi:10.1371/journal.pone.0089288)
Supplement: Table S1 — Characteristics of included epidemiological studies exploring the association between soy isoflavone intake and breast cancer risk. (DOCX) [file pone.0089288.s001.docx]

**Table S2** **Characteristics of included epidemiological studies exploring the association between soy isoflavone intake and breast cancer risk.**

| **First author (publication year)** | **Country or region** | **Study design** | **Population (cases/controls*)** | **Comparison**  **[method of measurement]** | **OR/RR(95%CI)** | **Adjustment factors**  **(matched by)** |
| --- | --- | --- | --- | --- | --- | --- |
| Laura N. Aderson et al.[1] (2013) | Canada | Population based case-control study  (ER+/PR+) | Premenopausal:  (473/1211)  Postmenopausal:  (959/2154) | Adult isoflavone intake (ug/day):  (3rd vs. 1st tertile)  [FFQ] | Premenopausal:  0.82(0.61-1.10)  Postmenopausal:  1.02(0.80-1.30) | Age, history of benign disease, dietary fiber intake, age at first live birth and family history of breast cancer. |
|  | Canada | Population based case-control study  (ER-/PR-) | Premenopausal:  (184/1211)  Postmenopausal:  (314/2154) | Adult isoflavone intake (ug/day):  (3rd vs. 1st tertile)  [FFQ] | Premenopausal:  1.18(0.78-1.79)  Postmenopausal:  1.50(1.05-2.15) |  |
|  | Canada | Population based case-control study  (ER+/PR-) | Premenopausal:  (79/1211)  Postmenopausal:  (386/2154) | Adult isoflavone intake (ug/day):  (3rd vs. 1st tertile)  [FFQ] | Premenopausal:  1.12(0.60-2.07)  Postmenopausal:  1.08(0.77-1.53) |  |
| Aida Karina Zaineddin et al.[2](2012) | German | Population based case-control study | Postmenopausal  (2884/5509) | Consumption of soybeans(high vs. nonconsumers)  Low and high consumers of the soybeans were defined by the 50^th^ percentile of consumption among controls.  [FFQ] | Postmenopausal:  0.83(0.70-0.97) | BMI, education level, first-degree family history of breast cancer, history of benign breast disease, number of pregnancies, age at menarche, breastfeeding history, total number of mammograms, smoking habit, alcohol consumption, phytoestrogen supplement use, energy intake, fiber intake.  (age and study center) |
| Qiong Wang et al.[3] (2011) | China (Sichuan) | Hospital based case-control study | Premenopausal:  224 pairs  Postmenopausal:  176 pairs | Energy-adjusted daily intake of soy isoflavones levels (≥23.55mg/day vs. <9.95 mg/day)  [FFQ] | Premenopausal:  0.47(0.26-0.87)  Postmenopausal:  0.13(0.05-0.33) | BMI, smoking, total energy intake, total protein intake, total fat intake, age at menarche, age at first birth, no. of abortions, duration of breast feeding, and oral contraceptive ues.  (age(±2 years) and menopausal status) |
| Yan-yun Zhu et al.[4] (2011) | China (Guangzhou and Beijing) | Hospital based case-control study | Premenopausal  (78/97)  Post- or perimenopausal  (105/95) | Soy isoflavone intake (>28.83 mg/day vs. <7.56 mg/day)  [Interviewer administered FFQ] | **Soy isoflavone:**  Premenopausal:  0.66(0.31-1.07)  Post- or perimenopausal:  0.57(0.29-0.83) | Age, smoking, passive smoking, drinking, family history of cancer, history of breast disease, vegetables and fruit  (a five-year age groups) |
| Theodore M. Brasky et al. [5](2010) | America | Cohort study | Postmenopausal:  (880/34136) | Soy supplement (user vs. non-user)  [Questionnaire on supplement use] | Postmenopausal:  1.04(0.74-1.48) | Age, race, education, BMI, height, fruit consumption, vegetable consumption, alcohol consumption, physical activity, age at menarche, age at menopause, age at first birth, history of hysterectomy, years of hormone therapy, family history of breast cancer, history of benign breast biopsy, mammography, low-dose aspirin use, regular aspirin use, ibuprofen use, naproxen use, and use of multivitamins. |
| Ya Cho et al. [6](2010) | Korea | Hospital-based case-control study | Premenopausal:  (210/196)  Postmenopausal:  (148/164) | Isoflavones (mg / day)/total soy products (g /day)  (4th vs. 1st quartile)  [FFQ] | **Isoflavones:**  Premenopausal:  1.36(0.64-2.91) postmenopausal:  0.33(0.15-0.72)  **Total soy products:**  Premenopausal:  0.77(0.35-1.70) postmenopausal:  0.08(0.03-0.22) | Age, BMI, family history of breast cancer, current use of dietary supplements, education, occupation, smoking, alcohol intake, age at menarche, parity, total energy intake and postmenopausal hormone use for postmenopausal women. |
| Caixia Zhang et al.[7] (2010) | China  (Guangdong) | Hospital-based case-control study | Premenopausal:  (306/295)  Postmenopausal:  (132/143) | Soy isoflavone (>16.89mg/day vs.<3.26mg/day)  [Interviewer administered FFQ] | Premenopausal:  0.46(0.26-0.82)  Postmenopausal:  0.66(0.30-1.44) | Age at menarche, BMI, history of benign breast disease, mother/sister/daughter with breast cancer, physical activity, passive smoking, total energy, total vegetable, and total fruit intake. (age(5-year interval) and residence (rural/urban)) |
| Larissa A. korde et al. [8](2009) | America | Population-based case-control study | Premenopausal:  (438/684)  Postmenopausal:  (151/277) | Adult soy intake:  (3rd vs. 1st tertile)  [FFQ] | Premenopausal:  0.79(0.56-1.12)  Postmenopausal:  0.53(0.29,0.98) | Age at first live birth, parity, study site, ethnicity, age, age at menarche, family history of breast cancer, and personal history of benign breast disease.  (matched by study site, ethnicity, and year of birth(in 5-year groups)) |
| Sang-Ah Lee et al.[9] (2009) | China (Shanghai) | Cohort study | Premenopausal:  (305/not obtain)  Postmenopausal:  (289/not obtain)  (number of noncases: 72631) | Average isoflavone intake:  (5th vs. 1st quintile)  [FFQ] | Premenopausal:  0.44(0.26-0.73)  Postmenopausal:  1.09(0.78-1.52) | Age, education, physical activity, age at first live birth, BMI, season of recruitment, family history of breast cancer, and total energy intake. |
| Motoki Iwasaki et al.[10] (2009) | Japan, | Hospital-based case-control study | Premenopausal:  (178/137)  Postmenopausal:  (212/235) | Dietary isoflavone intake: (3rd tertile vs. 1st tertile)  [Interviewer administered FFQ] | Premenopausal:  1.35(0.72-2.54)  Postmenopausal:  0.62(0.38-1.01) | Age, area, number of births, family history of breast cancer, smoking status, moderate physical activity in the past 5 years, and vitamin supplement use.  (age(within 5 years) and ethnicity) |
|  | Brazil | Hospital-based case-control study | Premenopausal:  (25/24)  Postmenopausal:  (56/57) | Dietary isoflavone intake: (median 2 vs. median 1)  [Interviewer administered FFQ] | Premenopausal:  0.17(0.03-0.84)  Postmenopausal:  0.84(0.37-1.92) |  |
|  | Brazil | Hospital-based case-control study | Premenopausal:  (161/145)  Postmenopausal:  (218/234) | Dietary isoflavone intake: (consumers vs. non-consumers)  [Interviewer administered FFQ] | Premenopausal:  0.54(0.26-1.31)  Postmenopausal:  0.58(0.33-1.03) |  |
| Min Zhang et al.[11] (2009) | China (Hangzhou) | Hospital-based case-control study  (ER+) | Premenopausal:  (296/671)  Postmenopausal:  (135/338) | Total isoflavones: (mg/day) (>25.40 vs. <7.78)  (4th quartile vs. 1st quartile)  [FFQ] | **Total isoflavones:**  Premenopausal:  0.44(0.28-0.70)  Postmenopausal:  0.31(0.15-0.64) | Age at interview, residential area, education, BMI, age at menarche, oral contraceptive use, hormone replacement therapy, breast cancer in first degree relatives, alcohol consumption, tobacco smoking, passive smoking, tea drinking, physical activity, and total energy intake.  (age) |
|  | China (Hangzhou) | Hospital-based case-control study  (ER-) | Premenopausal:  (204/671)  Postmenopausal:  (121/338) | Total isoflavones: (mg/day) (>25.40 vs. <7.78)  (4th quartile vs. 1st quartile)  [FFQ] | **Total isoflavones:**  Premenopausal:  0.36(0.21-0.62)  Postmenopausal:  0.25(0.11-0.54) |  |
| Ruth C. Travis et al.[12] (2008) | Britain | Cohort study | (cases/person-years)  Premenopausal:  (196/22209)  Postmenopausal:  (290/10842) | Isoflavone intake (10+mg/day vs. <10mg/day)  [FFQ] | Premenopausal:  1.31(0.95-1.81)  Postmenopausal:  0.95(0.66-1.38) | Height, BMI, age at menarche, age at first birth, parity, alcohol consumption, daily energy intake and current HRT use. |
| Mi Kyung Kim et al.[13] (2008) | Korea | Hospital-based case-control study | Premenopausal:  235 pairs  Postmenopausal:  127 pairs | Soy protein(g/day)/total tofu intake(g/day) (5th quintile vs. <1st quintile)  [Interviewer administered FFQ] | **Soy protein:**  Premenopausal:  0.39(0.22-0.93)  Postmenopausal:  0.22(0.06-0.88)  **Total tofu intake:**  Premenopausal:  0.23(0.11-0.48)  Postmenopausal:  0.39(0.11-1.90) | Drinking, multivitamin use, number of children, breast feeding, quintile of carbohydrate intake, and quintile of energy, vitamin E, and folate.  (age (within 2 yr) and menopausal status) |
| AH Wu et al.[14] (2008) | Singapore | Cohort study | (cases/person-years)  Premenopausal:  (190/96605)  Postmenopausal:  (439/241568) | Isoflavones (mg1000kcal^-1^)(≥10.6mg vs. <10.6mg)  [FFQ] | Premenopausal:  1.04(0.77-1.40)  Postmenopausal:  0.74(0.61-0.90) | Age, years of interview, dialect, education, family history of breast cancer, parity, age when period became regular, BMI, and n-3 fatty acid. |
| Takeshi Suzuki et al.[15] (2008) | Japan | Population-based case-control study | Premenopausal:  (329/1645)  Postmenopausal:  (349/1745) | Soybean products (g/day) (3rd tertile vs. 1st tertile1)  [FFQ] | **Soybean products:**  Premenopausal:  0.74(0.54-1.02)  Postmenopausal:  0.84(0.61-1.15) | Drinking habit, smoking habit, BMI, regular exercise, family history of breast cancer, total nonalcohol energy intake, multivitamin use, age at menarche, parity, HRT use, referral pattern to hospital and age at menopause for postmenopausal women. (age (±0 years) and menopausal status) |
| Heather Ward et al.[16] (2008) | Britain | Nested case-control study | Premenopausal and perimenopausal:  (43/174)  Postmenopausal:  (194/778) | Total urinary isoflavones:  (not obtain)  [not obtain] | Premenopausal and perimenopausal:  1.30(1.04-1.64)  Postmenopausal:  1.01(0.96-1.13) | Age, weight, oral contraceptive use, menopausal hormone treatment, parity, menarche, breastfeeding, family history of breast cancer, daily intake of fat and energy, and batch. |
| Maria Hedelin et al.[17] (2008) | Sweden | Cohort study | (Case/person-years)  Age<50yΩ(premenopausal):  (494/405517)  Age>=50y (postmenopausal):  (520/183874) | Total isoflavonoids: (4th quartile vs. 1st quartile)  [Self-administered FFQ] | Age<50y (premenopausal):  1.04(0.81-1.34)  Age>=50y (postmenopausal):  0.93(0.73-1.18) | Age, BMI, oral contraceptives, age at first pregnancy, age at menarche, parity, cancer in sisters or mothers, and intake of total energy, alcohol, and saturated fat. |
| Michelle Cotterchio et al.[18] (2008) | Canada | Population-based case-control study | Premenopausal:  (930/1211)  Postmenopausal:  (2067/2154) | Isoflavone intake (ug/day):  (5th quintile vs. 1st quintile)  [Interviewer administered FFQ] | Premenopausal:  0.96(0.69-1.33)  Postmenopausal:  1.09(0.83-1.41) | Age, family history of breast cancer, history of benign breast disease, dietary fiber intake, age at first live birth. (controls were 1:1 frequency-matched to cases) |
| Martijn Verheus et al.[19] (2007) | Dutch | Nested case-control study | Pre-and perimenopausal:  (87 pairs)  Postmenopausal:  (296 pairs) | Plasma genistein:  (3rd vs. 1st tertile)  [Laboratory measurement plasma samples] | Pre-and perimenopausal+:  0.80(0.38-1.69)  Postmenopausal++:  0.69(0.45-1.04) | ++ Crude OR;  + ORs and 95%CIs adjusted for age at menarche.  (both matched by recruitment data(±6months)) |
| Brian N. Fink et al.[20] (2007) | America (Long Island) | Population-based case-control study | Premenopausal:  (457/487)  Postmenopausal:  (977/953) | Isoflavones intake: (5th vs. 1st quintile)  [Self-administered FFQ] | Premenopausal:  1.14(0.76-1.72)  Postmenopausal:  1.02(0.76-1.38) | Age and energy intake.  (frequency-matched by 5-year age groups) |
| Kazuko Nishio et al.[21] (2007) | Japan | Cohort study | Postmenopausal:  (92/22144) | Tofu (almost daily vs. ≤2 times/week)  [Self-administered FFQ] | Postmenopausal:  1.43(0.81-2.52) | Age, study area, family history of breast cancer, age at menarche, age at first birth, use of exogenous female hormone, smoking, consumption of green leafy vegetables, walking time, BMI, and total energy intake. |
| Min Hee Do et al.[22] (2007) | Korea | Hospital-based case-control study | Premenopausal:  (196/392)  Postmenopausal:  (163/316) | Total soy foods:  (high vs. low intake)  [Interviewer administered FFQ] | Premenopausal:  0.76(0.46-1.67)  Postmenopausal:  0.69(0.48-1.52) | Age, education, age at menarche, family history of breast cancer, age at first live birth, age at menopause, total duration of breast feeding, physical activity, total menstruation period, current BMI, alcohol consumption, cigarette smoking, frequency of exercise, total energy intake, total fat intake, total fruits intake, total vegetable intake, vitamin A,C,E and vitamin supplement |
| Marina S. Touillaud et al.[23] (2006) | France | Cohort study | Case/person-years:  Premenopausal:  (402/117652) | Total isoflavones: (4th quartile vs. 1st quartile)  [Diet record] | Premenopausal:  1.00(0.76-1.31) | Years of education, height, BMI, age at menarche, personal history of benign breast disease or lobular carcinoma in situ, family history of breast cancer in first- or second-degree relatives, lifetime use of oral contraceptive, age at first full-term pregnancy and parity, geographic area, alcohol consumption, and dietary energy intake from food. |
| K Hirose et al.[24] (2005) | Japan | Hospital-based case-control study | Premenopausal:  (79/414)  Postmenopausal:  (88/440) | Isoflavone intake/soybean products (3rd tertile vs. 1st tertile)  [FFQ] | **Isoflavone intake:**  Premenopausal:  0.44(0.22-0.89)  Postmenopausal:  0.58(0.30-1.10)  **Soybean products intake:**  Premenopausal:  0.53(0.27-1.04)  Postmenopausal:  0.70(0.37-1.33) | Age, motives for consultation, smoking, drinking, exercise, energy, family history, age at menarche, parity, age at first full-term pregnancy, BMI and age at menopause for postmenopausal women. |
| Clement A. Adebamowo et al.[25] (2005) | America | Cohort study | (Cases/ person-years)  Premenopausal: (710/706991) | Beans or lentils:  (2-4 servings /week vs. <1 serving/month)  [Self-administered FFQ] | Premenopausal:  0.76(0.57-1.00) | Age at menarche, parity and age at first birth, family history of breast cancer, history of benign breast disease, oral contraceptive use, alcohol consumption, energy intake, BMI, height, smoking habit, physical activity. |
| Jakob Linseisen et al.[26] (2004) | German | Population-based case-control study | Premenopausal:  (278/666) | IsoflavonoidsΨintake (4th vs. 1st quartile)  [Self-administered FFQ] | Premenopausal:  Isoflavone intake:  0.85(0.54-1.33) | First-degree family history of breast cancer, number of births, duration of breast-feeding, energy intake, BMI, alcohol consumption and education.  (exact age and study region) |
| Seiichiro Yamamoto et al.[27] (2003) | Japan | Cohort study | Premenopausal:  (89/9935)  Postmenopausal:  (87/11741) | Isoflavone consumption (mg/day) (4th vs. 1st quartile)  [Specific food Questionnaire] | Premenopausal:  0.66(0.25-1.70)  Postmenopausal:  0.32(0.14-0.71) | Age, age at menarche, number of pregnancies; age at first pregnancy; active and passive smoking; alcohol consumption; leisure-time physical activity; educational level; total energy; and meat, fish, vegetable, and fruit consumption. |
| Kaoru Hirose et al.[28] (2003) | Japan | Hospital-based case-control study | Premenopausal:  (1336/12003)  Postmenopausal:  (1049/7010) | Soybean curd (tofu): (≥5 times/week vs. 1-3 times/month) | Premenopausal:  0.84(0.67-1.04)  Postmenopausal:  0.83(0.65-1.05) | Age, visit year, family history, age at menarche, age at menopause, parity, age at first full-term pregnancy and BMI. |
| Pamela L. Horn-Ross et al.[29] (2001) | America | Population-based case-control study | Premenopausal:  (398/471)  Postmenopausal:  (826/1077) | Total isoflavone consumption(4th quartile vs. 1st quartile)  [FFQ] | Premenopausal:  1.20(0.75-2.00)  Postmenopausal:  0.96(0.71-1.30) | Age, race/ethnicity, age at menarche, parity, lactation, history of benign breast disease, family history of breast cancer; education; a composite variable including menopausal status, BMI, and HRT use; and daily caloric intake. |
| Xiao Ou Shu et al.[30] (2001) | China Shanghai | Population-based case-control study | Premenopausal:  (952/990)  Postmenopausal:  (501/562) | Soy foods (total, during adolescence) (5th quintile vs. 1st quintile)  [FFQ] | Premenopausal:  0.53(0.39-0.72)  Postmenopausal:  0.49(0.33-0.74) | Intake level of rice and wheat products, age, education, family history of breast cancer, history of breast fibroadenoma, WHR, age at menarche, physical activity, ever had live birth, and age at menopause.  (age (5-year interval).) |
| Den Tonkelaar et al.[31] (2001) | Dutch | Nested case-control study | Postmenopausal:  (88/268) | Urinary genistein: (3rd vs. 1st tertile)  [Laboratory test urinary samples] | Postmenopausal:  0.83(0.46-1.51) | Crude ORΦ |
| TJ Key et al.[32] (1999) | Japan | Cohort study | Cases/person-years：  Age ≤54 (premenopausal):  (150/184369)  Age >54  (postmenopausal):  (277/304620) | Tofu：  (≥5 times/week vs. ≤1 time/week)  [The mail survey questionnaire] | Age<50:  1.16(0.56-2.38)  Age≥50:  1.05(0.73-1.49) | Age, calendar period, city, and age at time of atom bombs, estimated radiation dose. |
| John S. Witte et al.[33] (1997) | America and Canada | Population-based case-control study (bilateral breast cancer) | Premenopausal:  (140/222) | Tofu(or soybean): (one serving per week vs. none)  [FFQ] | Premenopausal:  0.5(0.2-1.1) | Age, age at menarche, parity, oral contraceptive use, alcohol consumption, BMI, and energy.  (each case’s unaffected sister(s) served as matched control(s)) |
| Greenstein et al.[34]θ(1996) | Western | Cohort study | (cases/person-years)  Postmenopausal:  (1018/34388) | Soy or tofu: (consumers vs. non-consumers)  [not obtain] | Postmenopausal:  0.76(0.50-1.18) | “major breast cancer risk factors”(not specified). |
| Hin Peng Lee et al. [35](1992) | Singapore | Hospital-based case-control study | Premenopausal:  (109/207)  Postmenopausal:  (91/213) | Soy protein: (≥3.5 vs. < 1.6g/day)  [FFQ] | Premenopausal:  0.4(0.2-0.8)  Postmenopausal:  1.8(0.8-3.6) | Premenopausal: age, age at first birth  Postmenopausal: age, nulliparity, height, education, family history. |

* Controls represent No. of control subjects, if specified, they represent No. of person-years.

**Key et al [32]. did not provide numbers of cases by menopausal status. According to given numbers by age group, we assumed that 150 cases aged ≤54 years were premenopausal and 277 cases aged >54 years were postmenopausal.

θThe original study was reported only as published abstract. We extracted OR estimates from meta-analysis written vy Bruce J. Trock et al.[36] .

ΨSometimes isoflavones also referred to as isoflavonoids[37].

Ω Maria Hedelin et al. [74] separated premenopausal women from postmenopausal women based on the mean age at menopausal in Sweden (50 y).

ΦThe original study did adjust for age, height, weight, parity, age at menopause, history of benign breast disease, family history estrogen supplements, smoking, and laboratory run, but in multivariate model these factors did not affect the results substantially (<10% change in ORs), so they were abandoned for adjustment in final multivariate model.

Abbreviations: BMI for Body Mass Index, HRT for Hormone Replacement Therapy, FFQ for food frequency questionnaire.

**Reference：**

1. Anderson LN, Cotterchio M, Boucher BA, Kreiger N (2013) Phytoestrogen intake from foods, during adolescence and adulthood, and risk of breast cancer by estrogen and progesterone receptor tumor subgroup among Ontario women. Int J Cancer 132: 1683-1692.

2. Zaineddin AK, Buck K, Vrieling A, Heinz J, Flesch-Janys D, et al. (2012) The association between dietary lignans, phytoestrogen-rich foods, and fiber intake and postmenopausal breast cancer risk: a German case-control study. Nutr Cancer 64: 652-665.

3. Wang Q, Li H, Tao P, Wang YP, Yuan P, et al. (2011) Soy isoflavones, CYP1A1, CYP1B1, and COMT polymorphisms, and breast cancer: a case-control study in southwestern China. DNA Cell Biol 30: 585-595.

4. Zhu Y-y, Zhou L, Jiao S-c, Xu L-z (2011) Relationship Between Soy Food Intake and Breast Cancer in China. 12: 4.

5. Brasky TM, Lampe JW, Potter JD, Patterson RE, White E (2010) Specialty supplements and breast cancer risk in the VITamins And Lifestyle (VITAL) Cohort. Cancer Epidemiol Biomarkers Prev 19: 1696-1708.

6. Cho YA, Kim J, Park KS, Lim SY, Shin A, et al. (2010) Effect of dietary soy intake on breast cancer risk according to menopause and hormone receptor status. Eur J Clin Nutr 64: 924-932.

7. Zhang C, Ho SC, Lin F, Cheng S, Fu J, et al. (2010) Soy product and isoflavone intake and breast cancer risk defined by hormone receptor status. Cancer Sci 101: 501-507.

8. Korde LA, Wu AH, Fears T, Nomura AM, West DW, et al. (2009) Childhood soy intake and breast cancer risk in Asian American women. Cancer Epidemiol Biomarkers Prev 18: 1050-1059.

9. Lee SA, Shu XO, Li H, Yang G, Cai H, et al. (2009) Adolescent and adult soy food intake and breast cancer risk: results from the Shanghai Women's Health Study. Am J Clin Nutr 89: 1920-1926.

10. Iwasaki M, Hamada GS, Nishimoto IN, Netto MM, Motola J, Jr., et al. (2009) Dietary isoflavone intake and breast cancer risk in case-control studies in Japanese, Japanese Brazilians, and non-Japanese Brazilians. Breast Cancer Res Treat 116: 401-411.

11. Zhang M, Yang H, Holman CD (2009) Dietary intake of isoflavones and breast cancer risk by estrogen and progesterone receptor status. Breast Cancer Res Treat 118: 553-563.

12. Travis RC, Allen NE, Appleby PN, Spencer EA, Roddam AW, et al. (2008) A prospective study of vegetarianism and isoflavone intake in relation to breast cancer risk in British women. Int J Cancer 122: 705-710.

13. Kim MK, Kim JH, Nam SJ, Ryu S, Kong G (2008) Dietary intake of soy protein and tofu in association with breast cancer risk based on a case-control study. Nutr Cancer 60: 568-576.

14. Wu AH, Koh WP, Wang R, Lee HP, Yu MC (2008) Soy intake and breast cancer risk in Singapore Chinese Health Study. Br J Cancer 99: 196-200.

15. Suzuki T, Matsuo K, Tsunoda N, Hirose K, Hiraki A, et al. (2008) Effect of soybean on breast cancer according to receptor status: a case-control study in Japan. Int J Cancer 123: 1674-1680.

16. Ward H, Chapelais G, Kuhnle GG, Luben R, Khaw KT, et al. (2008) Breast cancer risk in relation to urinary and serum biomarkers of phytoestrogen exposure in the European Prospective into Cancer-Norfolk cohort study. Breast Cancer Res 10: R32.

17. Hedelin M, Lof M, Olsson M, Adlercreutz H, Sandin S, et al. (2008) Dietary phytoestrogens are not associated with risk of overall breast cancer but diets rich in coumestrol are inversely associated with risk of estrogen receptor and progesterone receptor negative breast tumors in Swedish women. J Nutr 138: 938-945.

18. Cotterchio M, Boucher BA, Kreiger N, Mills CA, Thompson LU (2008) Dietary phytoestrogen intake--lignans and isoflavones--and breast cancer risk (Canada). Cancer Causes Control 19: 259-272.

19. Verheus M, van Gils CH, Keinan-Boker L, Grace PB, Bingham SA, et al. (2007) Plasma phytoestrogens and subsequent breast cancer risk. J Clin Oncol 25: 648-655.

20. Fink BN, Steck SE, Wolff MS, Britton JA, Kabat GC, et al. (2007) Dietary flavonoid intake and breast cancer risk among women on Long Island. Am J Epidemiol 165: 514-523.

21. Nishio K, Niwa Y, Toyoshima H, Tamakoshi K, Kondo T, et al. (2007) Consumption of soy foods and the risk of breast cancer: findings from the Japan Collaborative Cohort (JACC) Study. Cancer Causes Control 18: 801-808.

22. Do MH, Lee SS, Kim JY, Jung PJ, Lee MH (2007) Fruits, vegetables, soy foods and breast cancer in pre- and postmenopausal Korean women: a case-control study. Int J Vitam Nutr Res 77: 130-141.

23. Touillaud MS, Thiebaut ACM, Niravong M, Boutron-Ruault M-C, Clavel-Chapelon F (2006) No association between dietary phytoestrogens and risk of premenopausal breast cancer in a french cohort study. Cancer Epidemiology Biomarkers & Prevention 15: 2574-2576.

24. Hirose K, Imaeda N, Tokudome Y, Goto C, Wakai K, et al. (2005) Soybean products and reduction of breast cancer risk: a case-control study in Japan. Br J Cancer 93: 15-22.

25. Adebamowo CA, Cho E, Sampson L, Katan MB, Spiegelman D, et al. (2005) Dietary flavonols and flavonol-rich foods intake and the risk of breast cancer. Int J Cancer 114: 628-633.

26. Linseisen J, Piller R, Hermann S, Chang-Claude J (2004) Dietary phytoestrogen intake and premenopausal breast cancer risk in a German case-control study. Int J Cancer 110: 284-290.

27. Yamamoto S, Sobue T, Kobayashi M, Sasaki S, Tsugane S (2003) Soy, isoflavones, and breast cancer risk in Japan. J Natl Cancer Inst 95: 906-913.

28. Hirose K, Takezaki T, Hamajima N, Miura S, Tajima K (2003) Dietary factors protective against breast cancer in Japanese premenopausal and postmenopausal women. Int J Cancer 107: 276-282.

29. Horn-Ross PL, John EM, Lee M, Stewart SL, Koo J, et al. (2001) Phytoestrogen consumption and breast cancer risk in a multiethnic population: the Bay Area Breast Cancer Study. Am J Epidemiol 154: 434-441.

30. Shu XO, Jin F, Dai Q, Wen W, Potter JD, et al. (2001) Soyfood intake during adolescence and subsequent risk of breast cancer among Chinese women. Cancer Epidemiol Biomarkers Prev 10: 483-488.

31. den Tonkelaar I, Keinan-Boker L, Veer PV, Arts CJ, Adlercreutz H, et al. (2001) Urinary phytoestrogens and postmenopausal breast cancer risk. Cancer Epidemiol Biomarkers Prev 10: 223-228.

32. Key TJ, Sharp GB, Appleby PN, Beral V, Goodman MT, et al. (1999) Soya foods and breast cancer risk: a prospective study in Hiroshima and Nagasaki, Japan. Br J Cancer 81: 1248-1256.

33. Witte JS, Ursin G, Siemiatycki J, Thompson WD, Paganini-Hill A, et al. (1997) Diet and premenopausal bilateral breast cancer: a case-control study. Breast Cancer Res Treat 42: 243-251.

34. Greenstein J, Kushi L, Zheng W, Fee R, Campbell D, et al. (1996) Risk of breast cancer associated with intake of specific foods and food groups. American Journal of Epidemiology 143: S36-S36.

35. Lee HP, Gourley L, Duffy SW, Esteve J, Lee J, et al. (1992) Risk factors for breast cancer by age and menopausal status: a case-control study in Singapore. Cancer Causes Control 3: 313-322.

36. Trock BJ, Hilakivi-Clarke L, Clarke R (2006) Meta-analysis of soy intake and breast cancer risk. J Natl Cancer Inst 98: 459-471.

37. Kelly GE, Nelson C, Waring MA, Joannou GE, Reeder AY (1993) Metabolites of dietary (soya) isoflavones in human urine. Clin Chim Acta 223: 9-22.
