# Supplementary material for: Association between Soy Isoflavone Intake and Breast Cancer Risk for Pre- and Post-Menopausal Women: A Meta-Analysis of Epidemiological Studies
Source: PLoS One. 2014 Feb 20;9(2):e89288. doi: 10.1371/journal.pone.0089288 (PMC3930722; doi:10.1371/journal.pone.0089288)
Supplement: Forest Plots S1 — The word file presents six forest plots for subgroup analyses among premenopausal women, and in each analysis, studies were further stratified by study design, meanwhile, heterogeneity statistics for each stratified analysis were listed below each forest plot. (DOCX) [file pone.0089288.s005.docx]

Following are six forest plots for subgroup analyses among premenopausal women, and in each analysis, studies were further stratified by study design.

It is important to note that in each plot, 0 stands for retrospective case-control study, and 1 stands for cohort or nested case-control study. The results of homogeneity test for each stratified analysis were presented below the figure. Relative weights are indicated by the area of square. Horizontal lines represent 95% confidence intervals for the odds ratios.


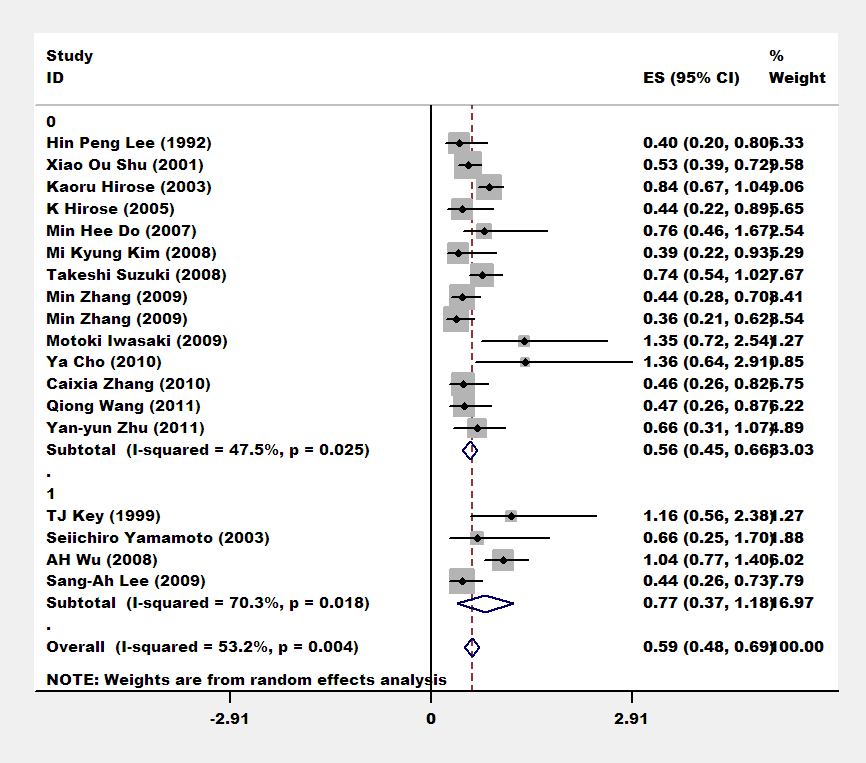


**Figure Asian** Associations between soy isoflavone intake and breast cancer risk in studies carried out in Asian countries among premenopausal women.

Heterogeneity of ORs for case-control studies: *χ^2^*(*df*)=24.76(13); *P*=0.025; *I^2^*(%)=47.5

Heterogeneity of ORs for cohort studies: *χ^2^*(*df*)=10.09(3); *P*=0.018*; I^2^*(%)=70.3


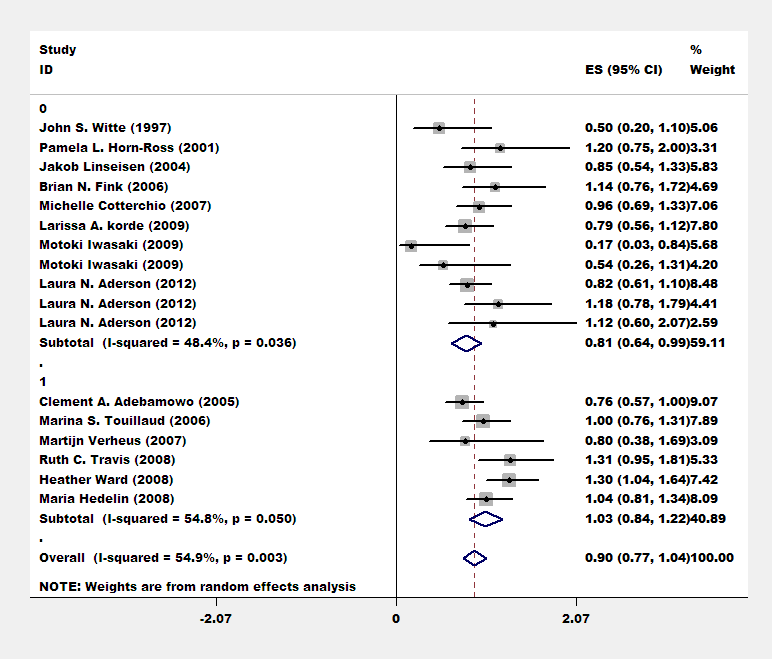


**Figure western** Associations between soy isoflavone intake and breast cancer risk in studies carried out in western countries among premenopausal women.

Heterogeneity of ORs for case-control studies: *χ^2^*(*df*)=19.39(10); *P*=0.036; *I^2^*(%)=48.4

Heterogeneity of ORs for cohort studies: *χ^2^*(*df*)=11.07(5); *P*=0.050*; I^2^*(%)=54.8


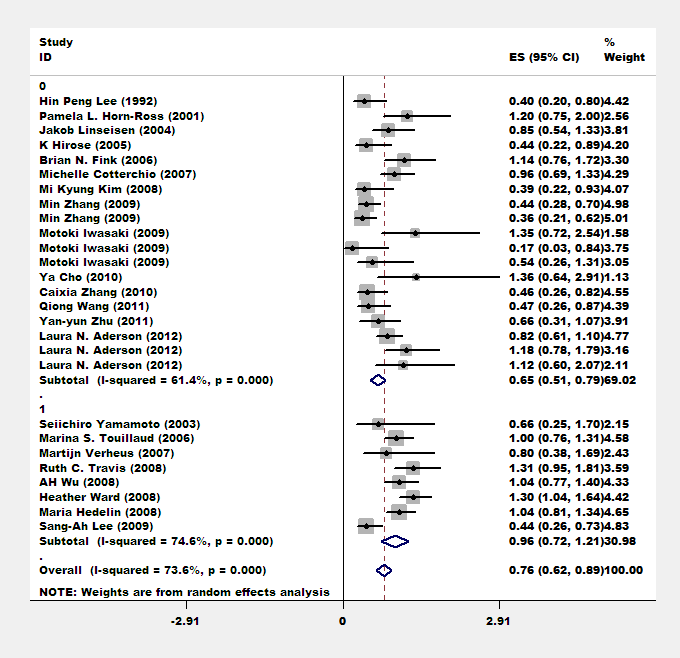


**Figure** **Soy isoflavone/ protein** Associations between soy isoflavone intake and breast cancer risk in studies used soy isoflavone or soy protein as intake measurement among premenopausal women.

Heterogeneity of ORs for case-control studies: *χ^2^*(*df*)=46.58(18); *P*=0.000; *I^2^*(%)=61.4

Heterogeneity of ORs for cohort studies: *χ^2^*(*df*)=27.61(7); *P*=0.000*; I^2^*(%)=74.6


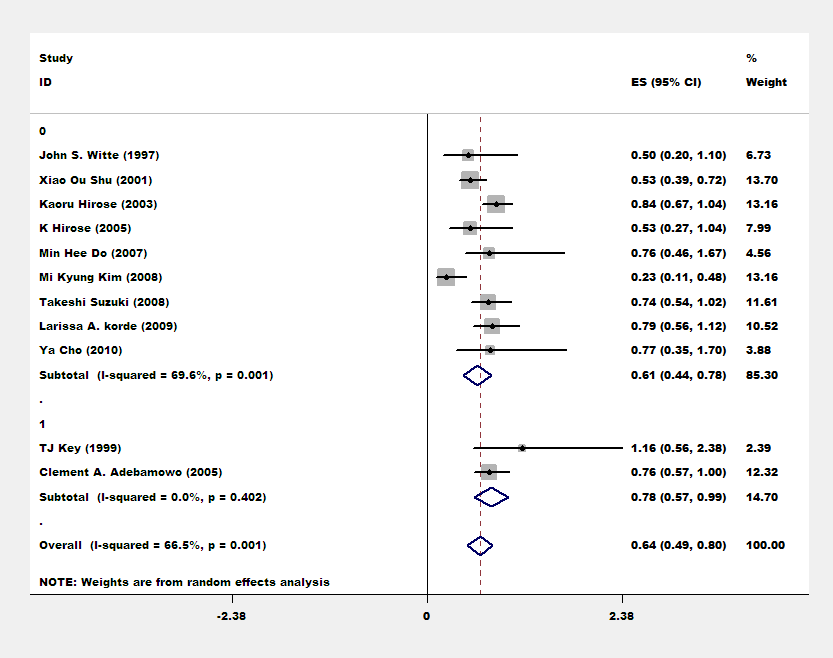


**Figure** **Soy bean/ soy products (foods)** Associations between soy isoflavone intake and breast cancer risk in studies used soy bean or soy products (foods) as intake measurement among premenopausal women.

Heterogeneity of ORs for case-control studies: *χ^2^*(*df*)=26.35(8); *P*=0.001; *I^2^*(%)=69.6

Heterogeneity of ORs for cohort studies: *χ^2^*(*df*)=0.70(1); *P*=0.402*; I^2^*(%)=0.0


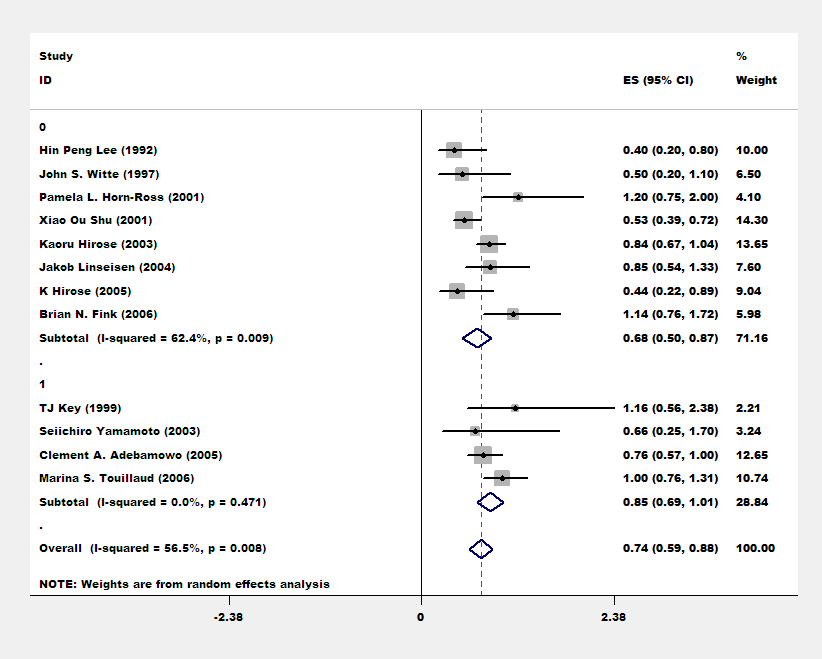


**Figure** **Earlier time** Associations between soy isoflavone intake and breast cancer risk in earlier studies (before 2006) among premenopausal women.

Heterogeneity of ORs for case-control studies: *χ^2^*(*df*)=18.64(7); *P*=0.009; *I^2^*(%)=62.4

Heterogeneity of ORs for cohort studies: *χ^2^*(*df*)=2.53(3); *P*=0.471*; I^2^*(%)=0.0


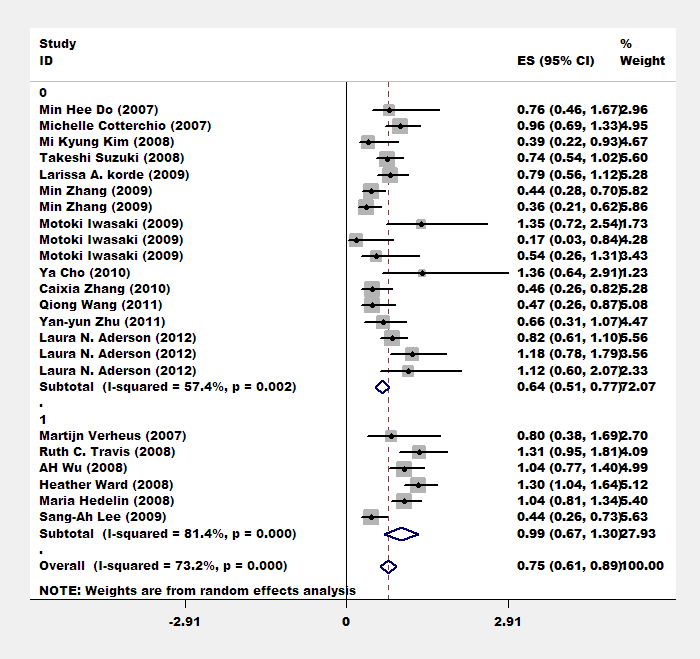


**Figure Later time** Associations between soy isoflavone intake and breast cancer risk in later studies (from 2006 to January 2013) among premenopausal women.

Heterogeneity of ORs for case-control studies: *χ^2^*(*df*)=37.59(16); *P*=0.002; *I^2^*(%)=57.4

Heterogeneity of ORs for cohort studies: *χ^2^*(*df*)=26.82(5); *P*=0.000*; I^2^*(%)=81.4
